# Supplementary material for: Antioxidant Constituents and Activities of the Pulp with Skin of Korean Tomato Cultivars
Source: Molecules. 2022 Dec 9;27(24):8741. doi: 10.3390/molecules27248741 (PMC9786122; doi:10.3390/molecules27248741)
Supplement: Supplementary file 1 [file molecules-27-08741-s001.zip › molecules-2054217-supplementary.pdf]

Supplementary Materials

Antioxidant Constituents and Activities of the Pulp with Skin of Korean Tomato Cultivars

Dong-Min Kang [1], Ji-Min Kwon [1], Woo-Jin Jeong [1], Yu Jin Jung [2,3], Kwon Kyoo Kang [2,3,*] and Mi-Jeong Ahn [1,*]

[1] College of Pharmacy and Research Institute of Pharmaceutical Sciences, Gyeongsang National University, Jinju 52828, Republic of Korea

[2] Division of Horticultural Biotechnology, Hankyong National University, Anseong 17579, Republic of Korea

[3] Institute of Genetic Engineering, Hankyong National University, Anseong 17579, Republic of Korea

* Correspondence: kykang@hknu.ac.kr (K.K.K.); amj5812@gnu.ac.kr (M.-J.A.); Tel.: +82-55-772-2425 (M.-J.A.)

Figure S1. LC chromatograms of the lipophilic extracts from thirteen tomato cultivars (450 nm).

Figure S2. LC chromatograms of the lipophilic extracts from five tomato cultivars (450 nm).

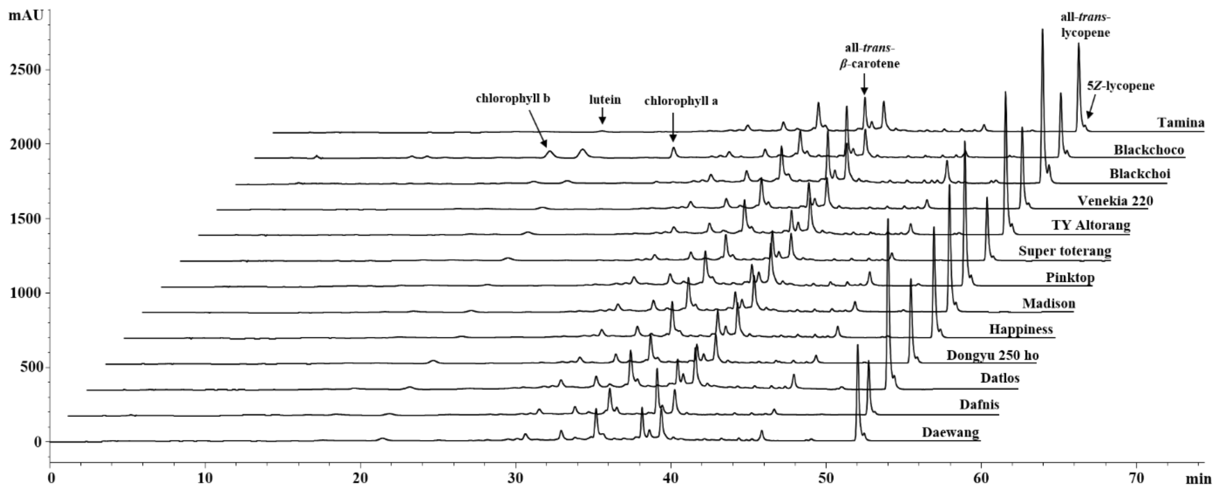

Figure S1. LC chromatograms of the lipophilic extracts from thirteen tomato cultivars (450 nm).

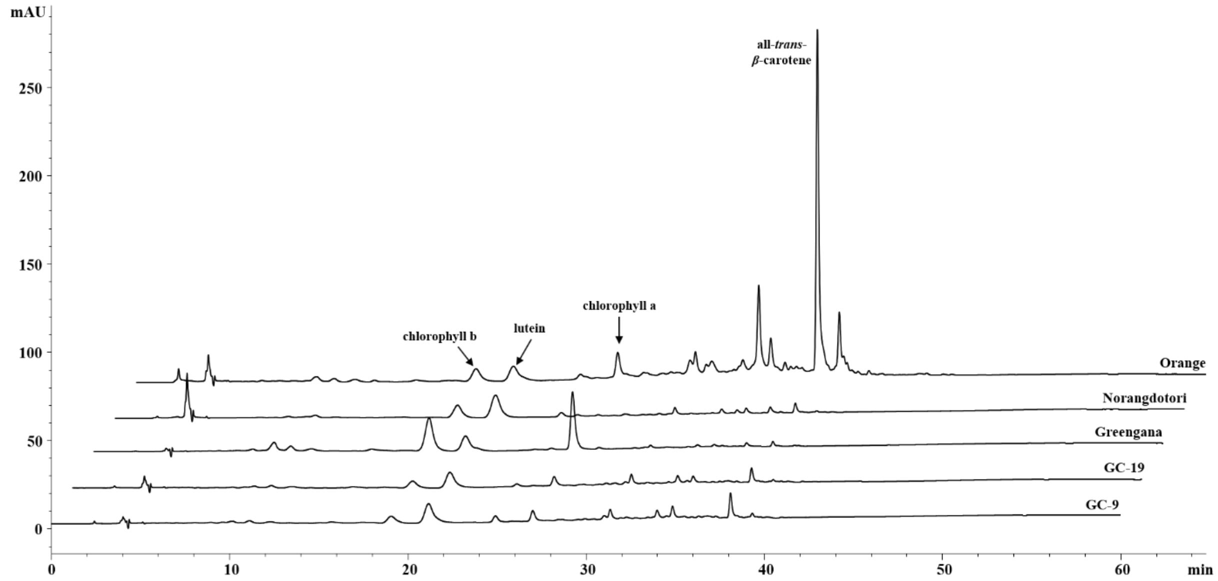

Figure S2. LC chromatograms of the lipophilic extracts from five tomato cultivars (450 nm).
